# Galveston County Medical Society

**Published:** 1893-12

**Authors:** David Cerna

**Affiliations:** Secretary


**Galveston County Medical Society.**—The following are the newly elected officers of the Galveston County Medical Society, for the year 1893–1894: President, Dr. Wm. Keiller; 1st Vice-President, Dr. A. F. Sampson; 2d Vice.President, Dr. James E. Thompson; Treasurer, Dr. W. H. Baldinger; Secretary; Dr. David Cerna. Executive Committee, Drs. A. J. Smith, R. C. Hodges and E. Randall.

At the last meeting of the Society, held November 20th, 1893, Dr. John B. Haden read a very interesting paper, entitled ''The Visual Field and the Perimeter,'' which was extensively discussed by Drs. Smith, Hodges, Thompson and Keiller. The paper will be sent to the JOURNAL for publication.

On motion of Dr. A. J. Smith, a committee was appointed, with full power to act, to confer with the medical fraternity of Houston and vicinity toward the formation of a District Medical Association, with which the Galveston County Medical Society shall be in affiliation. The committee thus appointed consists of Drs. A. J. Smith, R. J. Hodges and J. E. Thompson.

DAVID CERNA, M. D., Secretary.

